# Three novel *ANO5* missense mutations in Caucasian and Chinese families and sporadic cases with gnathodiaphyseal dysplasia

**DOI:** 10.1038/srep40935

**Published:** 2017-02-08

**Authors:** Lingling Jin, Yi Liu, Fanyue Sun, Michael T. Collins, Keith Blackwell, Albert S. Woo, Ernst J. Reichenberger, Ying Hu

**Affiliations:** 1 Beijing Institute of Dental Research, Beijing Stomatological Hospital, Capital Medical University, Beijing, China; 2 Department of Maxillofacial Surgery, Beijing Stomatological Hospital, Capital Medical University, Beijing, China; 3 Center for Regenerative Medicine and Skeletal Development, Department of Reconstructive Sciences, University of Connecticut Health, Farmington, CT, 06030, USA; 4 National Institute of Dental and Craniofacial Research (NIDCR), Bethesda, MD, 20892, USA; 5 Department of Head and Neck Surgery, UCLA, Los Angeles, CA, 90095, USA; 6 Division of Plastic Craniofacial and Pediatric Surgery, Brown University Warren Alpert Medical School, Providence, RI, 02903, USA

## Abstract

Gnathodiaphyseal dysplasia (GDD; MIM#166260) is an autosomal dominant syndrome with characteristic cemento-osseous lesions of jawbones, bone fragility, and diaphyseal sclerosis of tubular bones. To date, only five mutations in the proposed calcium-activated chloride channel *ANO5/TMEM16E* gene have been identified. In this study, we describe two families and two singular patients with three new mutations. One Caucasian family with seven affected members exhibited frequent bone fractures and florid osseous dysplasia (p.Cys356Tyr), while one Chinese family with two affected members suffered from cementoma and purulent osteomyelitis (p.Cys360Tyr). In addition, two different novel mutations (p.Gly518Glu and p.Arg215Gly) were identified in sporadic patients without family history. *In vitro* studies overexpressing GDD mutations (p.Cys356Tyr and p.Cys360Tyr) showed significantly reduced ANO5 protein. It appears that all GDD mutations known so far locate in an extracellular domain following the first transmembrane domain or in the 4^th^ putative transmembrane domain. Both wild-type and mutant ANO5 protein localize to the endoplasmic reticulum. After *Ano5* gene knock-down with shRNA in MC3T3-E1 osteoblast precursors we saw elevated expression of osteoblast-related genes such as *Col1a1, osteocalcin, osterix* and *Runx2* as well as increased mineral nodule formation in differentiating cells. Our data suggest that ANO5 plays a role in osteoblast differentiation.

Gnathodiaphyseal dysplasia (GDD; MIM#166260) is an extremely rare skeletal bone disorder involving lesions of the mandible that are consistent with florid osseous dysplasia, combined with a complex skeletal phenotype of bone fragility, cortical thickening and sclerosis of diaphyses of tubular bones^1^. GDD had previously been named osteogenesis imperfecta with unusual skeletal lesions or gnatho-diaphyseal sclerosis and was first described in a large Japanese family including 21 patients exhibiting frequent bone fractures in adolescence and purulent osteomyelitis of the jaws during adult life^2^. While bone fragility and jaw lesions in some patients become obvious during adolescence, other patients may experience characteristic symptoms at birth or within the first months of life. GDD shares clinical and pathological features of syndromes involving fibro-osseous jaw lesions, most notably fibrous dysplasia (FD) and McCune-Albright syndrome (MAS). However, specific clinical, histological, and genetic characteristics suggest that GDD is as a distinct pathological entity^2^,^3^. FD and MAS are caused by activating missense mutations of the GNAS1 (α-stimulating guanine nucleotide binding protein 1)^3^,^4^.

GDD is inherited as an autosomal dominant trait or occurs sporadically and was first mapped to an 8.7 cM interval on chromosome 11q14.3–15.1 in a family previously described by Akasaka^5^. Subsequently, three mutations were identified in exon 11 in codon 356 (p.Cys356Arg, p.Cys356Gly and p.Cys356Tyr)^6^,^7^,^8^,^9^. Another missense mutation in exon 15 of *ANO5* was found in an Italian family (p.Thr513Ile)^10^ and more recently a p.Ser500Phe mutation in a single patient with GDD^11^. The gene responsible for GDD (*GDD1, TMEM16E, ANOCTAMIN5*, ANO5; MIM#608662) is a member of the *TMEM16* gene family of calcium-activated chloride channels^12^. *ANO5* encodes for a 913 amino-acid protein and belongs to a large family of transmembrane proteins which share a common predicted eight-transmembrane topology with N-and C-terminal cytoplasmic tails. The biochemical functions of ANO5 and the molecular pathophysiology of *ANO5* mutations leading to GDD have not been fully elucidated.

Here we report two families of Caucasian and Chinese origin with autosomal dominant GDD caused by a p.Cys356Tyr mutation in *ANO5* and a novel p.Cys360Tyr mutation, respectively as well as two novel heterozygous missense mutations (p.Gly518Glu and p.Arg215Gly) in two unrelated patients without family history. We describe the clinical features of the probands in detail as well as stimulatory effects on osteoblastogenesis by knocking down ANO5 in a pre-osteoblastic cell line and study the effects of p.Cys356Tyr and p.Cys360Tyr ANO5 mutations on protein expression.

## Results

### Clinical evaluation

**Figure 1:**
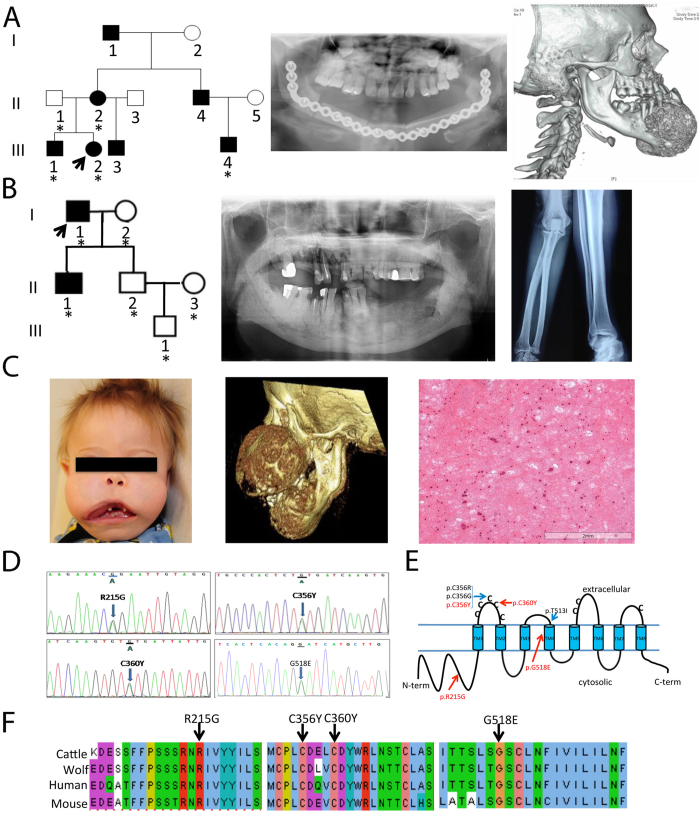
Mutation analysis of probands with familial or sporadic GDD. **(A)** Family 1 (Caucasian): Five individuals have been recruited (asterisk). Radiograph of mandible of proband after surgery and CT showing prior mandibular overgrowth and patchy sclerotic maxillary alveolus. **(B)** Family 2 (Han Chinese): Six individuals recruited including the proband (arrow) and his affected son. Orthopantogram showing overgrowth of both jaws with cotton-wool-like pattern in the alveolar regions. Radiograph of diaphyseal cortical thickening of tubular bones. **(C)** Sporadic patient 1 with enlargement of the maxilla and mandible. CT scan showing a symmetric expansion in anterior-posterior dimension. Histological findings of the jaw lesion exhibiting cementum-rich florid osseous dysplasia with fibrous proliferation of variable cellularity. **(D)** Electropherograms of mutations identified in Family 1 (c.1067 G > A; p.Cys356Tyr), Family 2 (c.1079 G > A; p.Cys360Tyr), sporadic patient 1 (c.1553 G > A; p.Gly518Glu) and sporadic patient 2 (c.643 A > G (p.Arg215Gly). **(E)** Schematic of *ANO5* protein. Previously reported GDD mutations are located in the first extra cellular domain and the 4^th^ transmembrane domain. Three novel mutations from this study are indicated in red. **(F)** Interspecies sequence alignment for ANO5 showing conservation of mutant residues (black arrows) in cattle (Bos taurus, NP_001161878.1), wolf (Canis lupus familiaris, NP_001161885.1), human (Homo sapiens, NP_998764.1) and mouse (Mus musculus, NP_808362.2).

The proband in Family 1 (Fig. 1A and Table 1) is a 15-year-old Caucasian female who presented with a 3- to 4-year history of a slowly enlarging chin. Computed tomography (CT) scans revealed a 7.1 × 5.6 × 5.5 cm anterior mandibular mass with a mixed lytic and sclerotic appearance. There was also a diffuse patchy sclerotic appearance of the maxillary alveolus, extending into the maxillary sinuses (Fig. 1A). A mandibular biopsy was consistent with juvenile florid osseous dysplasia, psammomatoid type. The patient underwent an angle-to-angle segmental resection of the mandible and one year later, a sublabial approach for bilateral partial maxillectomy was performed and mandibular hardware placed previously was removed to allow for unimpeded growth of her reconstructed jaw. All of the fibular and mandibular osteotomies were found to be well healed. Her past medical history was significant for multiple prior bone fractures, including a nasal fracture, a finger fracture, and 3 separate right ankle fractures. At the age of 1 year, she underwent surgery of correction of bilateral nasolacrimal duct obstruction. Family history was significant for a mandibular tumor that developed in her mother at the age of 21. The tumor was excised. Her 12-year-old brother was recently found to have jaw lesions on the basis of a panoramic radiograph. Numerous maternal relatives had a history of bone fractures, including her half-brother, her uncle, her grandfather, and her mother’s nephew. Prior to molecular diagnosis, the mother of the proband was diagnosed with polyostotic fibrous dysplasia. She had suffered repeated fractures. A tibia-fibula fracture at age 43 was complicated by osteomyelitis and skin necrosis and resulted in a lasting infected non-union wound. The patient underwent below-knee amputation. The mother’s parents were both unaffected and not tested for *ANO5* variants.

The Proband in Family 2 (Fig. 1B) is a 73-year-old Han Chinese male with a more than 30-year history of propensity for jaw infections. He was diagnosed with cementoma ten years ago and since then often suffered from purulent osteomyelitis-like symptoms including purulent discharge from gums, tooth mobility, loss of teeth, and insufficient healing after dental extractions. Panoramic radiological examinations showed multiple lobular or amorphous radiopacities in the tooth-bearing segments of the maxilla and mandible, and a cotton-wool-like pattern in the alveolar regions (Fig. 1B). He underwent surgical revision of tooth extraction sites and a subtotal mandibulectomy at the age of 62 years. Although he did not experience frequent fractures, his radiographs showed gross thickening of the diaphyseal cortices of long bones with narrow medullary canals (Fig. 1B). Histopathological examination of jaw lesions revealed a pattern similar to cemento-osseous dysplasia showing rounded calcified cementum-like structures.

Sporadic Patient 1 (Fig. 1C) is a four-year-old Caucasian male who had already suffered from bilateral femur fractures by 20 weeks prenatally and was diagnosed with osteopenia, rib and femur fractures and lacunar skull deformity (Lueckenschaedel). By the end of the first year of life he had multiple fractures of his femurs and compression fractures of lumbar vertebrae. Born with normal facial appearance, he developed maxillary and mandibular enlargements at 2 months of age and by 1 year of age was no longer able to close his mouth. CT scans documented a symmetric anterior-posterior expansion of the maxilla from 2.8 to 5.1 cm over 4 months (Fig. 1C). Debulking of the maxillary and mandibular masses was performed. The fibro-osseous lesion was described as cementum-rich and florid (Fig. 1C), containing fibrous proliferation with variable cellularity. Since then, he has undergone additional debulkings of mandibular masses and suffered fractures of femurs, fingers, toe and right parietal bone. The osteopenia is being treated with pamidronate.

Sporadic Patient 2 was previously reported by Riminucci^1^. He presented at the age of 13 months with bilateral relatively symmetric, expansile lesions in the maxillary bone associated with a sinus infection. Radiographic survey showed lytic lesions of the mandible. Histology revealed large eosinophilic masses of cementum-like material interspersed in fibrous background. Bowing and cortical thickening of diaphyseal regions of tibias and fibulae were present. The patient sustained five fractures, all occurring as a result of minimal trauma. The patient died from complications related to GDD.

### Molecular analysis

Mutation analysis of the patients in our study revealed three novel heterozygous mutations in exons 7, 11 and 15 of *ANO5*. A recently described c.1067 G > A transition (p.Cys356Tyr) cosegregated with the disease in Caucasian family 1 (Fig. 1D). Another missense mutation, c.1079 G > A, causing a p.Cys360Tyr mutation was found in the two affected members of a Chinese family. Sporadic patient 1 had a c.1553 G > A transition (Gly518Glu) in exon 15. Finally, a c.643 A > G mutation (p.Arg215Gly) was identified in sporadic patient 2. The parents of both sporadic patients were clinically and genotypically unaffected. These four *ANO5* (TMEM16E) variants in GDD patients were absent in dbSNP, 1000Genomes, the Exome Variant Server (~6500 exomes) and the ExAC database. Positions of mutations in ANO5 are shown in Fig. 1E. These mutations, which are defined as ‘probably damaging’ by PolyPhen-2 and ‘damaging’ by SIFT, are located in phylogenetically highly conserved regions (Fig. 1F).

### Low expression of mutant ANO5 protein

**Figure 2:**
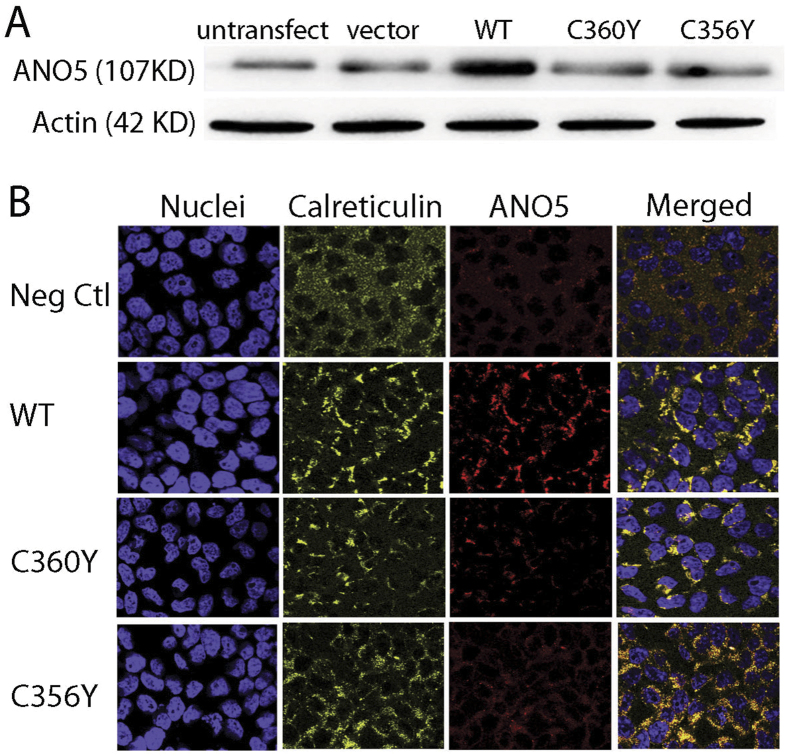
Expression of the hANO5 protein in HEK293 cells. **(A)** Western blot showing expression of endogenous ANO5 protein (untransfected and vector control), wild-type and mutant hANO5 (p.C356Y and p.C360Y) proteins. Mutant protein shows little or no expression. **(B)** Cellular localization of wild-type and mutant hANO5 proteins (red). Costaining with antibody against the calreticulin endoplasmic reticulum (ER)-specific marker (yellow) shows co-localization with the hANO5 protein (red).

To test for *in vitro* effects of GDD mutations we expressed 2 mutant ANO5 proteins (TMEM16Egdd(356Y) and TMEM16Egdd(360Y)) in HEK293 cells in a pEZ-M61 vector. The protein expression levels were much below that of wild-type TMEM16E (Fig. 2A). However, copy numbers of the mutant constructs were 51% (C356Y) and 107% (C360Y) higher compared to the wt construct copy number (data not shown). Immunofluorescent staining of transfected cells with ANO5 antibodies showed that the ANO5 protein co-localizes with calreticulin, an endoplasmic reticulum-specific marker expressed in the perinuclear area and that cells overexpressing mutant ANO5 protein exhibit reduced fluorescent intensities compared to wild-type ANO5 (Fig. 2B).

### *ANO5* knock-down promotes osteoblastogenesis

**Figure 3:**
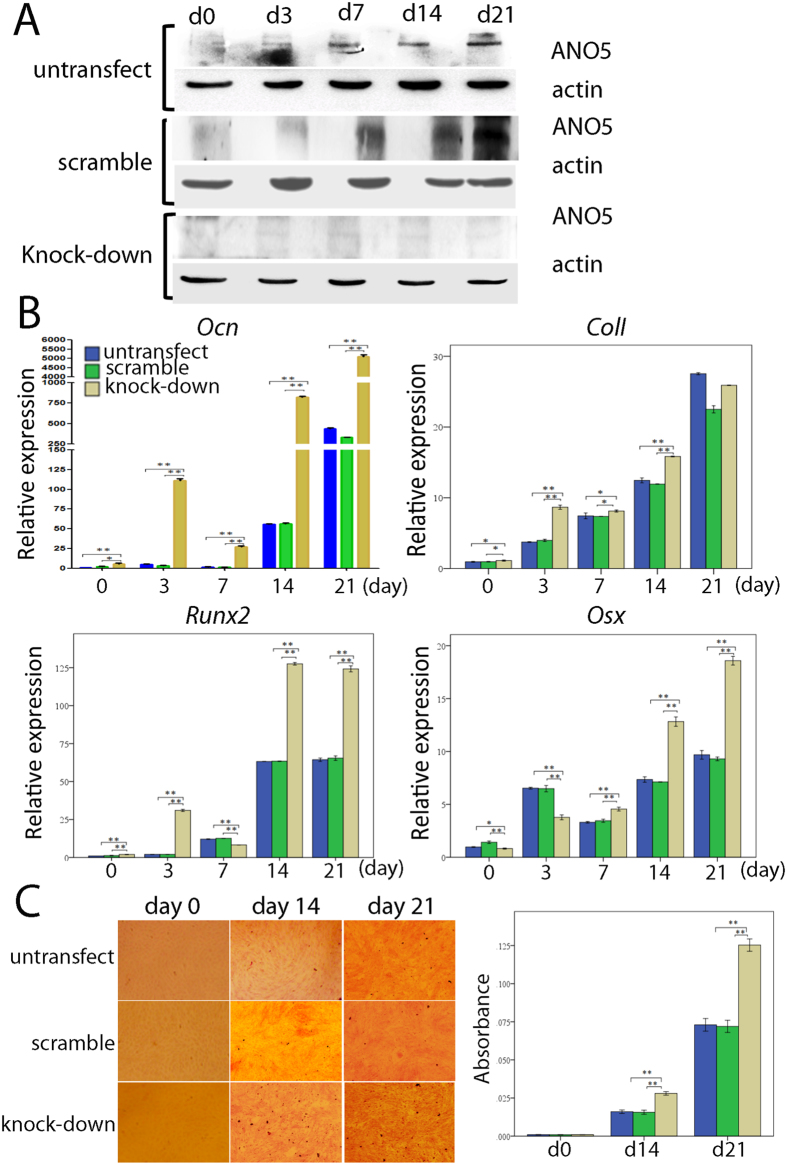
*Ano5* gene knock-down with shRNA. **(A)** Western blot of MC3T3-E1 cultures transfected with control or *Ano5*-specific shRNAs. Amounts of endogenous ANO5 protein increase over cell culture periods day 0 to day 21 in untransfected cell cultures (top row) and in cultures transfected with scrambled shRNA (middle row). Knock-down with *Ano5*-specific shRNAs is approximately 80% efficient (bottom row). **(B)** qPCR showing increasing *Ocn, Cola1, Runx2* and *osterix* in knock-down MC3T3-E1 cultures compared to control groups. Relative expression to GAPDH. **(C)** Matrix and mineral formation in MC3T3-E1 cell cultures after *ANO5* knock-down. Cells stained for alizarin red S. Mineral nodule formation is enhanced at days 14 and 21. Significant difference: *p < 0.05; **p < 0.01.

MC3T3-E1 Subclone 14 cells were transfected with control or *ANO5*-specific shRNAs. Resistant colonies were counted after two weeks of selection and the knock-down efficiency (approximately 80%) was verified by Western blotting (Fig. 3A). Subsequently, cells were cultured in osteogenic media and harvested after 0, 3, 7, 14 and 21 days. We detected significant differences in mRNA expression for osteoblast differentiation markers *osteocalcin (Ocn*), *Col1a1, Runx2* and *osterix* by quantitative PCR in *Ano5* knock-down cells compared to scrambled control shRNA (random RNA sequence) (Fig. 3B). *Ocn* expression increased since day 3 of culture, while saturation for collagen I (*Col1a1*) expression was seen after day 14. *Runx2* was also increased at day 3 in knock-down cells and again after day 14. The late osteoblast differentiation marker *osterix* increased after day 7 in culture suggesting increased numbers of mature osteoblasts in the culture. *Runx2* is expressed during early stages of osteoblast differentiation and then again at a late stage. Alizarin red staining at culture days 14 and 21 showed increased mineral nodule formation in *Ano5* knock-down cultures (Fig. 3C). The expression of endogenous ANO5 protein in MC3T3-E1 cells increased consistently during the course of osteoblastic differentiation over 21 days (Fig. 3A). MC3T3-E1 Subclone14 cells are widely used for osteoblast differentiation studies^13^,^14^.

## Discussion

Hallmarks of gnathodiaphyseal dysplasia are the development of cemento-osseous lesions of the mandible and increased bone fragility. However, there appears to be a wide age range in the onset of fibrous lesions of the mandible and severity of fractures. The mandibular mass of the proband from Family 1 developed around age 12 years and her fractures began to occur around the age of 7. The onset of fractures appears to be more common in the second decade of life^2^,^15^,^16^. The proband’s mother developed a lesion that was initially described as “ossifying fibroma”, but should be better termed as florid osseous dysplasia, at age 21 years and her brother was first diagnosed at age 12 years. Onset of osseous lesions of other patients with cysteine 356 mutations in the literature occurred at age 6^7^ and at age 3, respectively^8^,^15^. Multiple fractures and bowing of tibiae were already present at age 6. The proband of Family 2 suffered from jaw infections for more than 30 years. After diagnosis of cementoma at the age of 62, he had resection of the mandibular mass which necessitated partial excision of the mandible due to osteomyelitis and purulent exudates from continuous inflammatory reactions. While he did not experience frequent fractures, his radiographs showed gross thickening of diaphyseal cortices of long bones with narrow medullary canals. At age 43 his son, who is not as severely affected as his father, was initially diagnosed with fibrous dysplasia in the mandible which shares histopathologic similarity with GDD. He did not suffer from abnormal bone fractures. While some individuals with GDD who have facial abnormalities may not develop any bone fractures, other members of the same family may develop fractures in the second decade of life or late in life^10^. Early onset of bone fragility at 2 or 3 years of age has been reported^3^,^15^ and neonatal fractures were described in the patient presented by Levin *et al*.^17^. Interestingly, the sporadic patient 1 described here experienced bilateral femur fractures at prenatal age of 20 weeks and the sporadic patient 2 described by Riminucci and colleagues^1^ suffered 5 fractures between the age of 1 and 3 years.

A number of GDD patients develop osteomyelitis in the oral cavity with an increased propensity for bacterial infections, resulting in the secretion of purulent exudate (pus) from infected lesions^2^,^6^,^10^ or osteomyelitis^3^,^16^. Histopathological descriptions of tissue from GDD lesions note fibrous tissue with irregular acellular mineralized masses and small rounded spherical mineralized bodies (psammomatoid bodies)^1^,^3^,^10^,^15^,^18^. However, similar structures were also found in a patient with familial cementoma with jaw lesion and extragnathic polyostotic long bone lesions with fragile bones^19^, which may belong to the same spectrum of disorders as GDD.

Previously five mutations in the *ANO5* gene had been identified for GDD which affect cysteine 356 (p.Cys356Gly and p.Cys356Arg), threonine 513 (p.Thr513Ile) and serine 500 (p.Ser500Phe)^6^,^10^,^11^. In this study, we report novel heterozygous mutations in *ANO5* in a Chinese Family 2 (c.1079 G > A; p.Cys360Tyr) with characteristic features of GDD. Sporadic patient 1 had a transition (c.1553 G > A) leading to a p.Gly518Glu mutation. We also found a novel p.Arg215Gly mutation in sporadic patient 2 who had been clinically and phenotypically described in detail by Riminucci and colleagues^1^. The mutation in Caucasian family 1 (c.1067 G > A; p.Cys356Tyr) has recently been identified in three other families^7^,^8^,^9^. These four mutations were not present in publicly available databases which currently include data for more than 6000 genomes (ExAc) and prediction programs SIFT and PolyPhen 2.0 suggested the variants to be potentially damaging. All four mutations locate in phylogenetically highly conserved regions of ANO5. The mutation in sporadic patient 1 is either a *de novo* mutation or due to germline mosaicism. The mutations described here affect amino acids Cys356, Cys360, Arg215 and Gly518 indicating that Cys356 and Cys360 are within a domain with a critical function since the previously known mutations had been found at position Cys356 in the putatively first extracellular loop^6^ and Gly518 is only 5 amino acids distal of a known Thr513 mutation^10^ in transmembrane domain 4 (Fig. 1E). The p.Arg215Gly mutation is situated in a putative intracellular region. There is insufficient knowledge on the functions of ANO5 domains.

The *ANO5* gene encodes for a protein which contains eight transmembrane domains with the N- and C- termini located in the cytosol. Eight cysteine residues at positions 342, 353, 356, 360, 369, 601, 606, and 804 in the putative extracellular loops of human ANO5 are conserved in humans, teleost and insect species^6^ and may be important for the folding of the ANO5 protein by participating in intrachain bonds. Taken together, these data suggest that cysteines 356 and 360 are vital residues and that they may affect the function of ANO5. However, clinical symptoms between GDD families with Cys356 and Cys360 mutations described here are not identical and the potential for differences in pathology based on the mutation should be taken into account. Disturbance of transmembrane domains is almost always disruptive to protein function and replacing the polar threonine 513 with a hydrophobic isoleucine and exchanging the neutral glycine 518 with an acidic glutamic acid appears to damage the structure or function of ANO5 sufficiently to result in a GDD phenotype.

ANO5 belongs to a large family of transmembrane glycoproteins found in all eukaryotes and includes at least 10 members in mammalians. During early embryogenesis, ANO5 is expressed in myotomal and sclerotomal somites and at a later stage is abundant in cardiac and skeletal muscle as well as in chondrocytes and osteoblasts in mice. This suggests that ANO5 plays a key role in muscular/skeletal development and metabolism^20^. While some mutations in *ANO5* cause autosomal dominant gnathodiaphyseal dysplasia, at least 11 recessive mutations are known to cause limb-girdle muscular dystrophy (LGMD2L)^21^ and at least 2 recessive mutations are responsible for Miyoshi myopathy^22^. While ANO5-related muscular dystrophies have been relatively well researched, the molecular effects of ANO5 mutations on GDD are still unknown.

We performed preliminary functional studies *in vitro* to investigate effects of two mutations in ANO5 (C356Y and C360Y) during osteoblast formation. The expression of mutant protein was reduced as shown by Western blots and immunocytochemistry. Recent studies^23^ suggest that mutant ANO5 protein (C356G and C356R) folds with low efficiency and appears to be unstable and rapidly degraded via proteasomal degradation. Our study also provides evidence that overexpressed ANO5 is localized in the endoplasmic reticulum surrounding the nucleus. Membrane proteins localizing in the endoplasmic reticulum are often involved in protein processing, protein folding, or calcium homeostasis. ANO5 was suggested to act as calcium-activated chloride channel (CaCC) because of its structural similarity to other members of the ANO family (anion selective channels with eight transmembrane segments)^12^. However, according to a recent study, ANO5 protein does not appear to have chloride channel activity due to a threonine rather than a cysteine residue at amino acid 611 (UniProt: Q75V66) which is one of the three conserved cysteine residues in CaCC molecules (i.e., ANO1 and ANO2)^23^. While the low levels of ANO5 protein may suggest that haploinsufficiency could be the cause for the skeletal phenotype it is likely that mutant ANO5 or its partial degradation product elicits unwanted cellular responses that contribute to the GDD phenotype. The lack of an obvious skeletal phenotype in *Ano5* knock-out mice argue against a dose effect^24^. A broad phenotypic spectrum of limb girdle muscular dystrophy (LGMD2L) is caused by frameshift or point mutations in ANO5. Homozygous or compound heterozygous frameshift mutations that cause truncation of the protein close to the N-terminus or in the region where most GDD mutations have been found^25^ also argue against haploinsufficiency being the sole cause for the GDD phenotype.

Very little is known about the role of ANO5 in bone. A recent molecular study of ANO5 showed reduced expression of wild type and mutant ANO5 in cells, possibly because of protein instability and premature proteasomal degradation^23^. In order to investigate the association between ANO5 and bone, we knocked down the *Ano5* gene in a MC3T3-E1 preosteoblast cell line derived from mouse calvariae and evaluated changes in gene expression related to bone formation and the capacity for mineralization during osteoblast differentiation. The expression of *osteocalcin (Ocn*), *collagen type I (Col1a1*), *runt-related transcription factor 2 (Runx2*) and *osterix (Osx*) mRNA increased significantly after the knock-down of *Ano5*. Ocn is a marker of osteoblast activity and synthesized and secreted by mature mineralizing osteoblasts and osteocytes^26^. Runx2 and osterix are both required for the early and late stages of osteoblast differentiation, whereby Runx2 is a master regulator that acts upstream of osterix^27^. The transcription factors Runx2 and osterix can activate *Ocn* and *Col1a1* genes and involve transforming growth factor-β (TGF-β) and bone morphogenetic protein (BMP) pathways which have been implicated in regulation of osteoblast differentiation^28^. Interestingly, Camurati–Engelmann disease (CED) is a skeletal disorder which also manifests in diaphyseal hyperostosis of long bones caused by mutations in TGF-β1^29^ similar to the phenotype seen in GDD Family 2. Therefore, we speculate that ANO5 may regulate osteoblastogenesis and bone deposition via the TGF-β axis.

In our study, the mineralization properties of MC3T3-E1 cells, where the *Ano5* gene was knocked down, enhanced during osteoblastic differentiation (Fig. 3C), which is in line with a recent report of hypermineralization in a GDD patient^11^. These findings suggest that ANO5 may act as a negative regulator of mineralization and that reduced protein levels due to *ANO5* mutations or interactions of instable mutant ANO5 proteins may result in abnormal bone deposition or remodeling with over-mineralization, hyperostosis and brittle bones. We found that endogenous ANO5 expression is up-regulated during normal osteoblastic differentiation of this cell line.

In summary, our findings strongly indicate the association of ANO5 mutations with GDD, and we show that there is considerable clinical variability in patients, even within one family. These differences in expressivity can currently not be explained due to the limited number of patients available for phenotypic/genetic studies. However, potential modifier gene mutations in COL5A1 have recently been suggested for two affected siblings with GDD^8^. It is likely that the ANO5 mutations have cell autonomous effects on other cell types that contribute to the fibro-osseous mandibular lesions and to the long bone phenotype. Additional functional studies are needed to explain the roles of ANO5 in bone architecture and fibroproliferation.

## Materials and Methods

### Subjects

This study was approved by the Institutional Review Board of University of Connecticut Health, Farmington, CT, USA, the NIDCR IRB, Bethesda, MD, USA and the Ethics Committee of Beijing Stomatological Hospital, Beijing, China. Written informed consent had been obtained from study participants or their guardians. All genetic and experimental methods were carried out in accordance with relevant guidelines and regulations. Affected individuals underwent thorough clinical diagnosis and previous medical records were analyzed. The non-consanguineous unaffected parents of sporadic patients had no family history of genetic or skeletal disorders.

### Mutation testing

Genomic DNA was extracted from blood (Beijing ComWin Biotech), buccal mucosa (Beijing Zoman Biotechnology), saliva (Oragene Saliva Kit, DNA Genotek) or cultured cells (Qiagen DNeasy kit). Primers for polymerase chain reaction (PCR) amplification were designed using primer 3 (http://bioinfo.ut.ee/primer3) (Supplemental Table 1). After PCR amplification, 22 exons and flanking intron-exon boundaries of the *ANO5* gene were Sanger sequenced using ABI PRISM 3730xl DNA Analyzers with standard protocol. Potential detrimental effects of missense variants were evaluated by *in silico* prediction tools PolyPhen2 and SIFT. Sequence alignment among different species was performed with ClustalW2.

### Osteoblast differentiation

MC3T3-E1 Subclone 14 cells were cultured in DMEM supplemented with 15% fetal bovine serum, 2 mmol/l glutamine, 100 U/ml penicillin, and 100 μg/ml streptomycin (Invitrogen). MC3T3-E1 cells were cultured in α-MEM containing 10% FBS, 100 IU/ml penicillin and 100 μg/ml streptomycin. On day 7, culture medium was supplemented with 50 μg/ml ascorbic acid, 8 mM β-glycerophosphate and 10^−8^ mol/ml dexamethasone to induce osteoblast differentiation. Cells were grown under normoxic conditions with 5% CO_2_ at 37 °C. Medium was changed every other day.

### Plasmid construction and mutagenesis

The cDNA encoding human ANO5 protein was cloned into a pEZ-M61 vector and ANO5 mutations C356Yand C360Y were created in pEZ-M61using a QuickChange II Site-Directed Mutagenesis kit (Agilent Technologies, Massy, France) with primers (C356Y: Forward 5′-ACTCTGTGATCAAGTGTATGATTATTGGAGACTAAATAG-3′; Reverse 5′-CTATTTAGTCTCCAATAATCATACACTTGATCACAGAGT-3′ and C360Y: Forward 5′-GATCATGTGCCCACTCTATGATCAAGTGTGTGATTATTG-3′; Reverse 5′-CAATAATCACACACTTGATCATAGAGTGGGCACATGATC-3′). Mutations were verified by Sanger sequencing.

### Cell transfection

HEK-293 cells were cultured in 96-well microplates containing 100 μl of antibiotic-free DMEM (Sigma) containing 10% fetal calf serum. After 6 hours, cells were transfected with plasmids carrying either the sequence for wild-type or mutant *ANO5*. For each well, 0.2 μg of total plasmid DNA and 0.2 μl of Lipofectamine 2000 (Life Technologies) were first pre-mixed in 50 μl of OPTI-MEM (Life Technologies) to generate transfection complexes at room temperature for 60 minutes and then added to the cells. After 24 hours, the medium was replaced with fresh culture medium plus antibiotics.

### Immunohistochemistry

Wild-type and mutant HEK-293 cells were plated and cultured until confluent. Antibodies against calreticulin were used to label endoplasmic reticulum (ER) (yellow) (Anti-Calreticulin antibody 1:100; Abcam, UK). Human ANO5 protein (red) was visualized by (Anti-anoctamin 5; 1:100; Abcam, UK). Nuclei were labeled by DAPI (SantaCruz) (blue).

### Western blotting

After 48 hours of transfection, cells were lysed in RIPA buffer containing a protease inhibitor cocktail (P2714; Sigma), centrifuged for 2 minutes at 10,000 × g. Denaturing of the protein lysate was done by adding 1X Laemmli buffer containing 5% β-mercaptoethanol and incubation at 99 °C for 10 minutes. Proteins were separated by 10% SDS-PAGE and transferred to a PVDF membrane using a semidry transfer apparatus (Bio-Rad). The membranes were incubated in blocking buffer (LI-COR Biosciences) for 1hr and incubated with primary polyclonal antibody against ANO5 (1:1000; Abcam) and calreticulin (1:5000; Abcam) in blocking buffer containing 0.1% Tween20 at 4 °C overnight. The blots were stripped and reprobed with monoclonal antibodies against β-actin (1:5000; Sigma) as an internal control. HRP-conjugated secondary mouse antibody (Amersham) at a dilution of 1:3000 and the ECL detection system (Amersham) was used.

### *Ano5* gene knock-down

To generate stable *Ano5*-depleted cell lines, short hairpin RNAs (shRNA) against *Ano5* mRNA were subcloned into the psi-LVRH1GP lentiviral transfer vector (GeneCopoeia, CN). For virus infection, MC3T3-E1 cells were plated overnight, infected with lentivirus in the presence of 8 μg/ml polybrene (GeneCopoeia, CN) for 12 h and cultured with basic medium. After 96 hours, cells were selected with 8.5 μg/mL puromycin for 96 hours and cultured in α-MEM supplemented with 4.25 μg/mL puromycin. A scrambled shRNA clone was purchased from GeneCopoeia (CN). The shRNA sequences for the *Ano5* target were: 5′-CCGACAACCACTACCTGA-3′ and 5′-CTCTACAAATGTGGTATGGC-3′.

### RNA analysis

Total RNA from MC3T3-E1 cells was extracted using TRIzol reagent (Invitrogen) according to the manufacturer’s instructions. RNA was treated with DNase I (Invitrogen) and cDNA was synthesized using Superscript II reverse transcriptase (Invitrogen). Real-time PCR was performed using a QuantiFast SYBR Green PCR kit (QIAGEN, Germany) in an ABI-7500 instrument (Applied Biosystems). Relative quantification of gene expression was determined by the ΔΔCt method and GAPDH was used for data normalization. PCR primer sequences are listed in Supplemental Table 2. Experiments were performed at least three times.

### Alizarin red staining

To quantitatively determine the calcium mineral content, MC3T3-E1 cell cultures were stained with alizarin red. Cells were fixed in 4% formalin for 30 minutes and then treated with alizarin red (Cyagen, CN) according to the manufacturer’s instructions. The mineralized nodule number was counted under an inverted phase contrast microscope (Nikon, JPN) and the volume of mineralization nodules was calculated with MATLAB software by counting the pixels. Cells were destained for 15 minutes with 1 ml dimethylcarbinol at room temperature and the absorbance was measured at 550 nm on a multiplate reader. The final calcium level in each group was normalized to the total protein concentration determined from a duplicate plate by BCA. The experiment was repeated at least three times.

## Additional Information

**How to cite this article**: Jin, L. *et al*. Three novel *ANO5* missense mutations in Caucasian and Chinese families and sporadic cases with gnathodiaphyseal dysplasia. *Sci. Rep.* **7**, 40935; doi: 10.1038/srep40935 (2017).

**Publisher's note:** Springer Nature remains neutral with regard to jurisdictional claims in published maps and institutional affiliations.

## Supplementary Material

Supplementary Information

## Figures and Tables

**Table 1:** Features of GDD patients.

	Proband Family 1	Proband Family 2	Singular Patient 1	Singular Patient 2
ANO5 mutation	c.1067 G > A p.Cys356Tyr	c.1079 G > A p.Cys360Tyr	c.1553 G > A p.Gly518Glu	c.643 A > G p.Arg215Gly
Current age	15	73	4	deceased
Onset of symptoms	7	43	20 weeks prenatal	13 months
Sex	Female	Male	Male	Male
Jaw lesions	Anterior mandibular mass, juvenile florid osseous dysplasia, psammomatoid type	Purulent osteomyelitis-like lesions, cementoma	Maxillary and mandibular enlargements	Bilateral symmetric expansile maxillary bone
Number of bone fractures	5	0	>7	5

## References

[b1] Riminucci M. . Gnathodiaphyseal dysplasia: a syndrome of fibro-osseous lesions of jawbones, bone fragility, and long bone bowing. J Bone Miner Res 16, 1710–1718, doi: 10.1359/jbmr.2001.16.9.1710 (2001). 11547842

[b2] Akasaka Y., Nakajima T., Koyama K., Furuya K. & Mitsuka Y. Familial cases of a new systemic bone disease, hereditary gnatho-diaphyseal sclerosis. Nihon Seikeigeka Gakkai zasshi 43, 381–394 (1969). 5816667

[b3] Nishimura G. . Fragile bone syndrome associated with craniognathic fibro-osseous lesions and abnormal modeling of the tubular bones: report of two cases and review of the literature. Skeletal Radiol 25, 717–722 (1996). 8958616 10.1007/s002560050167

[b4] Rao V. V., Schnittger S. & Hansmann I. G protein Gs alpha (GNAS 1), the probable candidate gene for Albright hereditary osteodystrophy, is assigned to human chromosome 20q12-q13.2. Genomics 10, 257–261 (1991). 1904395 10.1016/0888-7543(91)90508-c

[b5] Tsutsumi S. . Autosomal dominant gnathodiaphyseal dysplasia maps to chromosome 11p14.3-15.1. J Bone Miner Res 18, 413–418, doi: 10.1359/jbmr.2003.18.3.413 (2003). 12619924

[b6] Tsutsumi S. . The novel gene encoding a putative transmembrane protein is mutated in gnathodiaphyseal dysplasia (GDD). Am J Hum Genet 74, 1255–1261, doi: 10.1086/421527 (2004). 15124103 PMC1182089

[b7] Vengoechea J. & Carpenter L. Gnathodiaphyseal dysplasia presenting as polyostotic fibrous dysplasia. Am J Med Genet A 167, 1421–1422, doi: 10.1002/ajmg.a.36986 (2015). 25866257

[b8] Andreeva T. V. . Whole exome sequencing links dental tumor to an autosomal-dominant mutation in ANO5 gene associated with gnathodiaphyseal dysplasia and muscle dystrophies. Scientific reports 6, 26440, doi: 10.1038/srep26440 (2016). 27216912 PMC4877638

[b9] Duong H. A. . Gnathodiaphyseal dysplasia: report of a family with a novel mutation of the ANO5 gene. Oral surgery, oral medicine, oral pathology and oral radiology 121, e123–128, doi: 10.1016/j.oooo.2016.01.014 (2016). PMC4830924 27068316

[b10] Marconi C. . A novel missense mutation in ANO5/TMEM16E is causative for gnathodiaphyseal dyplasia in a large Italian pedigree. Eur J Hum Genet 21, 613–619, doi: 10.1038/ejhg.2012.224 (2013). 23047743 PMC3658193

[b11] Rolvien T. . A Novel ANO5 Mutation Causing Gnathodiaphyseal Dysplasia With High Bone Turnover Osteosclerosis. J Bone Miner Res, doi: 10.1002/jbmr.2980 (2016). 27541832

[b12] Schroeder B. C., Cheng T., Jan Y. N. & Jan L. Y. Expression cloning of TMEM16A as a calcium-activated chloride channel subunit. Cell 134, 1019–1029, doi: 10.1016/j.cell.2008.09.003 (2008). 18805094 PMC2651354

[b13] Wang D. . Isolation and characterization of MC3T3-E1 preosteoblast subclones with distinct *in vitro* and *in vivo* differentiation/mineralization potential. J Bone Miner Res 14, 893–903, doi: 10.1359/jbmr.1999.14.6.893 (1999). 10352097

[b14] Alford A. I., Terkhorn S. P., Reddy A. B. & Hankenson K. D. Thrombospondin-2 regulates matrix mineralization in MC3T3-E1 pre-osteoblasts. Bone 46, 464–471, doi: 10.1016/j.bone.2009.08.058 (2010). 19744582 PMC2818128

[b15] Roginsky V. V., Ivanov A. L. & Khonsari R. H. Recurring gnathodiaphyseal dysplasia in two Russian brothers. Int J Oral Maxillofac Surg 39, 397–401, doi: 10.1016/j.ijom.2009.11.008 (2010). 20005074

[b16] Ahluwalia J., Ly J. Q., Norman E., Costello R. F. Jr. & Beall D. P. Gnathodiaphyseal dysplasia. Clin. Imaging 31, 67–69, doi: 10.1016/j.clinimag.2006.07.003 (2007). 17189853

[b17] Levin L. S. . Osteogenesis imperfecta with unusual skeletal lesions: report of three families. Am J Med Genet 21, 257–269, doi: 10.1002/ajmg.1320210207 (1985). 4014312

[b18] Shigeishi H. . Amphiregulin induces proliferative activities in osseous dysplasia. J Dent Res 88, 563–568, doi: 10.1177/0022034509338253 (2009). 19587163

[b19] Rossbach H. C., Letson D., Lacson A., Ruas E. & Salazar P. Familial gigantiform cementoma with brittle bone disease, pathologic fractures, and osteosarcoma: a possible explanation of an ancient mystery. Pediatric blood & cancer 44, 390–396, doi: 10.1002/pbc.20253 (2005). 15602717

[b20] Mizuta K. . Molecular characterization of GDD1/TMEM16E, the gene product responsible for autosomal dominant gnathodiaphyseal dysplasia. Biochemical and biophysical research communications 357, 126–132, doi: 10.1016/j.bbrc.2007.03.108 (2007). 17418107

[b21] Penttila S. . Eight new mutations and the expanding phenotype variability in muscular dystrophy caused by ANO5. Neurology 78, 897–903, doi: 10.1212/WNL.0b013e31824c4682 (2012). 22402862

[b22] Bolduc V. . Recessive mutations in the putative calcium-activated chloride channel Anoctamin 5 cause proximal LGMD2L and distal MMD3 muscular dystrophies. Am J Hum Genet 86, 213–221, doi: 10.1016/j.ajhg.2009.12.013 (2010). 20096397 PMC2820170

[b23] Tran T. T. . TMEM16E (GDD1) exhibits protein instability and distinct characteristics in chloride channel/pore forming ability. J Cell Physiol 229, 181–190, doi: 10.1002/jcp.24431 (2014). 23843187

[b24] Xu J. . Genetic disruption of Ano5 in mice does not recapitulate human ANO5-deficient muscular dystrophy. Skeletal muscle 5, 43, doi: 10.1186/s13395-015-0069-z (2015). 26693275 PMC4685631

[b25] Savarese M. . Next generation sequencing on patients with LGMD and nonspecific myopathies: Findings associated with ANO5 mutations. Neuromuscul. Disord. 25, 533–541, doi: 10.1016/j.nmd.2015.03.011 (2015). 25891276 PMC4502439

[b26] Zhang Q., Riddle R. C. & Clemens T. L. Bone and the regulation of global energy balance. J. Intern. Med. 277, 681–689, doi: 10.1111/joim.12348 (2015). 25597336 PMC4446154

[b27] Kern B., Shen J., Starbuck M. & Karsenty G. Cbfa1 contributes to the osteoblast-specific expression of type I collagen genes. J Biol Chem 276, 7101–7107, doi: 10.1074/jbc.M006215200 (2001). 11106645

[b28] Chen G., Deng C. & Li Y. P. TGF-beta and BMP signaling in osteoblast differentiation and bone formation. Int J Biol Sci 8, 272–288, doi: 10.7150/ijbs.2929ijbsv08p0272 [pii] (2012). 22298955 PMC3269610

[b29] Campos-Xavier B. . Phenotypic variability at the TGF-beta1 locus in Camurati-Engelmann disease. Hum Genet 109, 653–658, doi: 10.1007/s00439-001-0644-8 (2001). 11810278

