# Supplementary material for: Three novel ANO5 missense mutations in Caucasian and Chinese families and sporadic cases with gnathodiaphyseal dysplasia
Source: Sci Rep. 2017 Feb 8;7:40935. doi: 10.1038/srep40935 (PMC5296836; doi:10.1038/srep40935)
Supplement: Supplementary Information [file srep40935-s1.pdf]

Three novel *ANO5* missense mutations in Caucasian and Chinese families and sporadic cases with gnathodiaphyseal dysplasia

Lingling Jin[1], Yi Liu[2], Fanyue Sun[3], Michael T. Collins[4], Keith Blackwell[5], Albert S. Woo[6], Ernst J. Reichenberger[3*], Ying Hu[1*]

Supplemental Table 1. Oligonucleotides used to sequence the *ANO5* gene.

Supplemental Table 2. Amplification primers for quantitative real-time PCR.

Table 1. Oligonucleotides used to sequence the *ANO5* gene

Exon	Forward(5'-3')	Reverse(5'-3')
1	AAAGAGGCGTGGAAACAGG	AGCGAACCTCCCTAGGACTC
2	TGCTTTTCCTGTTATAGGCAGTT	GGGAGCCTCAAAACAAATCA
3	CCCACAGGCACATTTACAGA	GCATGTCAAATAACTGAACTGGA
4	TTGCTTTCTTCCCATTTTTCT	ACAATGTTCCCCCAAATCAA
5	TCTTCCTCCTGAGTCATTATTGC	CACTTCAGGGCCTCTCACTT
6	GCAGAGAGCCATCCAGAGTC	ACCACAGGCCCTATCATCC
7	TTGAAAATGCTTTGATGTGTTTG	CACCTTGCAGAAATGGTACG
8	TTCCTGACAACCAAGAGAAGC	GGATTTTCCCACCATGAGAG
9	TTGCATTTTGAGATAGCTTTGTG	TCAGTTTAGCAACAACATTCTCC
10	AGAAAGCAGTGGAGCCAAAA	ACCAGGTGAGAGGCAACTGT
11	AAAGCTGGGCTCTGAAAACTC	ACCCCAAATTCCCATGAATA
12	TGTTCCTCTTGCAGCACTGA	TGGCCACTAATATGCACTCC
13	AGCCACCTGAAACATGTGAA	TTGCAGAATATTTTAAAGGCAAAA
14	GGCCTGGAGATTTCTCCTTC	GGGAGAGGGAAAGGAAAGAC
15	TGCCAGTGTTCATGTCTTGC	CTCCTGGAGACCTGATCCAA
16	TCCTAGGCTTTTCCAGTGGTT	TGAATCAGTGCTTGACACACA
17	AACCCTTCCAACCAAAACCT	AAACCTTGCTAGCCATGGAA
18	GCTTTGGCTGGTGTCATTTC	TGGAACAGGGAAATAGGGAAC
19	TGTGGATCACTCCAAAACGA	AAGAGTTTGCATGACATAGGTTTTT
20	TGTTTCAGGACAAAGACTTGC	TCTGAATCATGCAATGTGTGAA
21	TTTTTCTGATCAACTAGAAAGGTCTG	TTTCCTTTCCTTTTCTGTCACC
22	TCTTCCTTGCCTTTCTACCTCA	CCCAAGGCCAGTGTTTCTTA

Table 2. Amplification primers for quantitative real-time PCR

Gene	Forward(5'-3')	Reverse(5'-3')
Ocn	CTCACAGATGCCAAGCCCA	CCAAGGTAGCGCCGGAGT
Col1a1	TTCTCCTGGTAAAGATGGTGC	GGACCAGCATCACCTTTAACA
Runx2	GGCAAGATGAGCGACGTGAG	ATCTGACTCTGTCCTTGTGG
Osterix	CTGGCTAGGTGGTGGTCAG	GTAGGGAGCTGGGTTAAGG
Ano5	CATGGGAATCCGAGTGGATG	GTGGAATTCGTTGAGTAGGC
Gapdh	AGGTCGGTGTGAACGGATTTG	TGTAGACCATGTAGTTGAGGTCA
